# Supplementary material for: A lipid nanoparticle-based oligodendrocyte-specific mRNA therapy
Source: Mol Ther Nucleic Acids. 2024 Nov 5;35(4):102380. doi: 10.1016/j.omtn.2024.102380 (PMC11617236; doi:10.1016/j.omtn.2024.102380)
Supplement: Document S1. Table S1 [file mmc1.pdf]

OMTN, Volume *35*

Supplemental information

A lipid nanoparticle-based oligodendrocyte-specific mRNA therapy

Masanori Sawamura, Kiyoshi Tachikawa, Rie Hikawa, Hisako Akiyama, Seiji Kaji, Ken Yasuda, Angel I. Leu, Hyojung Hong, Rajesh Mukthavaram, Pad Chivukula, Hodaka Yamakado, Yoshio Hirabayashi, Ryosuke Takahashi, and Shu-ichi Matsuzawa

Table S1. Primer sequences used for qPCR

Gene	Forward primer	Reverse primer
GAPDH	GGT CGG AGT CAA CGG ATT TG	TCA GCC TTG ACG GTG CCA TG
LDLR	GAG AGC TTG TGC CGA GAT GTG	CCG CAG TTG TTA GTG CCA TCA
VLDLR	AGA AAA GCC AAA TGT GAA CCC T	CAC TGC CGT CAA CAC AGT CT
ApoER2	ATG AGT GAC GTG AAT CCA CCC	GTC CAG GGC GGA ATA TGA GAA

Movies S1. LUNAR® transfers EGFP mRNA in oligodendrocyte.

After treatment with LUNAR®-*EGFP* mRNA, EGFP is gradually expressed in oligodendrocytes.

Movies S2. LUNAR® does not transfers EGFP mRNA in neurons.

After treatment with LUNAR®-*EGFP* mRNA, EGFP is not expressed in neurons.

Movies S3. The motor phenotype of non-injected twitcher mice.

The non-injected twitcher mice present motor dysfunction and tremorous movements.

Movies S4. The motor phenotype of twitcher mice treated with LUNAR®-*GalC* mRNA.

LUNAR®-*GalC* mRNA ameliorates the motor abnormalities in twitcher mice.
