# Supplementary material for: A lipid nanoparticle-based oligodendrocyte-specific mRNA therapy
Source: Mol Ther Nucleic Acids. 2024 Nov 5;35(4):102380. doi: 10.1016/j.omtn.2024.102380 (PMC11617236; doi:10.1016/j.omtn.2024.102380)
Supplement: Document S2. Article plus supplemental information [file mmc6.pdf]

Molecular Therapy
Nucleic Acids
Original Article

# A lipid nanoparticle-based oligodendrocyte-specific mRNA therapy

Masanori Sawamura,[1] Kiyoshi Tachikawa,[3] Rie Hikawa,[1] Hisako Akiyama,[4,5] Seiji Kaji,[1] Ken Yasuda,[1] Angel I. Leu,[3] Hyojung Hong,[3] Rajesh Mukthavaram,[3] Pad Chivukula,[3] Hodaka Yamakado,[2] Yoshio Hirabayashi,[6,7] Ryosuke Takahashi,[1] and Shu-ichi Matsuzawa[2]

[1]Department of Neurology, Graduate School of Medicine, Kyoto University, Kyoto, Japan; [2]Department of Therapeutics for Multiple System Atrophy, Graduate School of Medicine, Kyoto University, Kyoto, Japan; [3]Arcturus Therapeutics, San Diego, CA, USA; [4]Juntendo Advanced Research Institute for Health Science, Juntendo University, Tokyo, Japan; [5]RIKEN Center for Brain Science, Wako, Saitama, Japan; [6]Institute for Environmental and Gender-Specific Medicine, Juntendo University Graduate School of Medicine, Chiba, Japan; [7]Cellular Informatics Laboratory, RIKEN, Wako, Saitama, Japan

**Despite the wide range of applications of mRNA therapies, major difficulties exist in the efficient delivery of mRNA into oligodendrocytes, a type of glial cell in the brain. Commonly used viral vectors are not efficient in transforming oligodendrocytes. In this study, we introduced mRNAs into oligodendrocytes with high efficiency and specificity using LUNAR lipid nanoparticles. The uptake of LUNAR lipid nanoparticles occurred via low-density lipoprotein receptors in the presence of apoprotein E. A single dose of LUNAR-human galactosylceramidase mRNA significantly improved phenotypes and survival of twitcher mice, a mouse model of Krabbe disease wherein oligodendrocytes are damaged by galactosylceramidase deficiency. This approach to mRNA therapeutics, combined with cell-specific nanocarriers, demonstrates remarkable potential for the treatment of neurological disorders associated with oligodendrocytes.**

## INTRODUCTION

Many remarkable developments have recently been reported in the field of nucleic acid medicines. For instance, nusinersen was approved by the US Food and Drug Administration as the first nucleic acid therapeutic for spinal muscular atrophy (SMA) and other intractable neurological disorders. Nusinersen has been shown to significantly improve motor function and lifespan in SMA patients.[1,2] Moreover, the development and rapidly advancing clinical application of COVID-19 vaccines have triggered great interest in mRNA-based therapy. Although mRNA therapies have a wide range of clinical applications, such as those in the treatment of various cancers, there exist major difficulties in achieving efficient delivery of mRNA, especially into the brain, due to the obstacles presented by the blood-brain barrier (BBB). Many nucleic acid therapeutics have been reported to have the capability to target neurons. However, nucleic acid therapeutics targeting other cell types, such as glial cells, have not been reported, and the cell specificity of mRNA delivery poses potential difficulties in clinical applications.

Nucleic acid therapeutics have been considered for the treatment of leukodystrophies, a group of hereditary neurological disorders, in which the white matter of the CNS is damaged due to the loss of myelin and the glial cells that it is produced from, namely oligodendrocytes, resulting in various symptoms such as cognitive dysfunction and gait disorders. Leukodystrophies include diseases such as Krabbe disease, heterozygous leukodystrophy, adrenoleukodystrophy, Alexander disease, and Pelizaeus-Merzbacher disease (PMD). Adrenoleukodystrophy and Krabbe disease have been reported to occur in 1 in 20,000 individuals and 1 in 100,000 individuals, respectively.[3,4] Both diseases are known to be fatal, and no fundamental treatments have been established for them.

The inefficiency of gene transfer to oligodendrocytes is one of the primary reasons that fundamental treatments using nucleic acid therapeutics have not been established for leukodystrophies, despite several attempts. Adeno-associated virus (AAV), a commonly used viral vector, has a low transforming efficiency and specificity for oligodendrocytes. Various methods have been implemented in attempts to improve the transforming efficiency of AAV. von Jonquieres et al. characterized the human myelin-associated glycoprotein promoter, which was used for reliable targeting of AAV-mediated transgene expression in oligodendrocytes *in vivo*.[5] Powell et al. characterized Olig001, a novel AAV with preferential oligodendrocyte tropism.[6] Li et al. reported that oligodendrocyte-specific *Plp1* gene suppression therapy involving the use of artificial microRNA with a *CNP* promoter in AAV could serve as a potential cure for PMD.[7] Georgiou et al. showed that Cx47 expression, specifically in oligodendrocytes, by means of an AAV with a myelin basic protein promoter could ameliorate pathological symptoms in PMD model mice.[8] Despite the dedicated efforts of researchers, highly

Received 12 April 2024; accepted 31 October 2024;
https://doi.org/10.1016/j.omtn.2024.102380.

**Correspondence:** Ryosuke Takahashi, Department of Neurology, Graduate School of Medicine, Kyoto University, Kyoto, Japan.
**E-mail:** ryosuket@kuhp.kyoto-u.ac.jp
**Correspondence:** Shu-ichi Matsuzawa, Department of Therapeutics for Multiple System Atrophy, Graduate School of Medicine, Kyoto University, Kyoto, Japan.
**E-mail:** matsuzawa.shuichi.4a@kyoto-u.ac.jp


© 2024 The Author(s).
Published by Elsevier Inc. on behalf of The American Society of Gene and Cell Therapy.
This is an open access article under the CC BY license (http://creativecommons.org/licenses/by/4.0/).

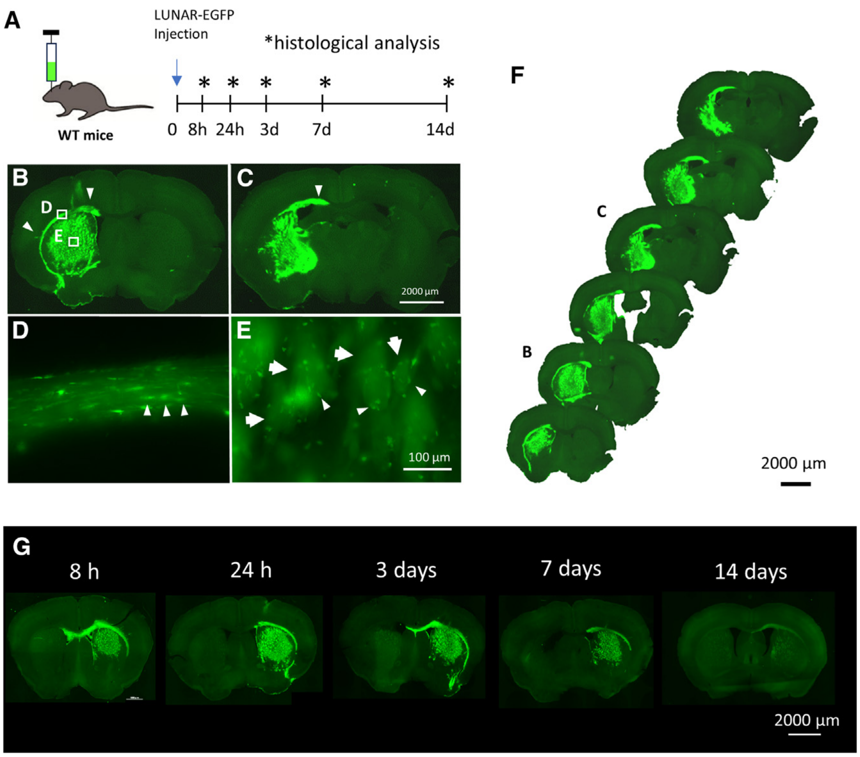


**Figure 1. LUNAR transfers EGFP mRNA in oligodendrocytes with high efficiency and specificity**

(A) Schematic representation of the experimental design. (B–F) Distribution pattern of EGFP transferred with LUNAR in mice after 24-h treatment with LUNAR-*EGFP* mRNA. High EGFP expression is observed in the white matter in injected mice (green color, arrowheads). Small cells in the corpus callosum (D) and striatum (E) shows EGFP expression. (G) The representative images of EGFP expression by LUNAR *EGFP* mRNA after 8 h–14 days in mice.

efficient gene delivery into oligodendrocytes remains challenging. Methods for gene delivery into oligodendrocytes have not been applied clinically, and the development of a new drug delivery system (DDS) for gene transfer into oligodendrocytes is highly sought after.

Several studies have reported the use of lipid nanoparticles (LNPs) functioning as nanocarriers of mRNA to be a DDS that is capable of efficiently crossing the BBB. However, the cell types in the brain associated with the uptake of these liposomes have not been extensively reported. Glial fibrillary acidic protein (GFAP) and SRY-box transcription factor $2^+$ cells show enhanced uptake of apoprotein E (ApoE)-modified liposomal particles.[9] However, it is still unclear whether other cell types, such as oligodendrocytes, are capable of the uptake of liposomes.

In the present study, we performed detailed histological analyses of brains injected with LUNAR, an LNP containing a class of proprietary ionizable amino lipids called ATX lipid, as a carrier of mRNA.[10,11] The ATX lipid contains an ionizable amino head group and a biodegradable lipid backbone. The ionizable amino head group renders the lipid with a pKa of <7. At acidic pH (e.g., pH 3.5), the amino group is protonated and interacts with the negatively charged RNA, thus forming nanoparticles and encapsulating the RNA. The pH sensitivity of the amino groups also allows for the protonation of lipids inside the endosome, thereby promoting interaction with oppositely charged anionic endosomal lipids, causing release of the RNA payload into the cytosol. It is reported that LUNAR was efficient and safe for systemic delivery for protein replacement therapy.

The present study showed LUNAR to be capable of delivering mRNA into oligodendrocytes with high efficiency and specificity. Moreover, we used LUNAR LNPs for the treatment of Krabbe disease model mice, an autosomal recessive inherited disease caused by a deficiency of the enzyme galactocerebrosidase (GALC), leading to the impairment of oligodendrocytes and Schwann cells, which results in demyelination, mental retardation, and movement disorders.[12–15] Our results might be valuable in the development of a nanocarrier combined with therapeutic mRNA that targets a specific cell type.

## RESULTS

### Expression pattern of EGFP proteins induced by LUNAR-*EGFP* mRNA

The particle sizes of LUNAR-*EGFP* and -*human GALC (hGALC)* mRNA were 80 and 92 nm, respectively. LUNAR-*EGFP* and -*hGALC* mRNA contained 2 mg/mL mRNA, and their encapsulation efficiency was 98.9% and 97.7%, respectively. To assess the distribution of mRNA transduced by LUNAR in the brain, we injected LUNAR-*EGFP* mRNA into the unilateral striatum of the brains of C57BL/6J mice. LUNAR-injected mice were sacrificed at the indicated time points, and their brain tissue was examined by histological analysis (Figure 1A). Results of the histological analysis showed that EGFP proteins were widely expressed mainly in the striatum 24 h after the treatment (Figures 1B–1F). In particular, EGFP was strongly expressed in small round cells in the corpus callosum (Figure 1D) and the white matter of the striatum (Figure 1E), suggesting EGFP expression in oligodendrocytes. EGFP expression in mice was observed after 8 h, peaked at 24 h–3 days, and decreased after 7 days (Figure 1G). To further analyze which cells specifically express EGFP proteins, we evaluated the percentage of EGFP-expressing cells that colocalized with cell-specific markers: neurons (NeuN), astrocytes (GFAP), microglia (Iba1), and oligodendrocytes (Olig2 or GST$\pi$). The expression of EGFP was seen to colocalize with the oligodendrocyte markers Olig2 or GST$\pi$, but not with NeuN, GFAP, or Iba1 (Figures 2A–2E).

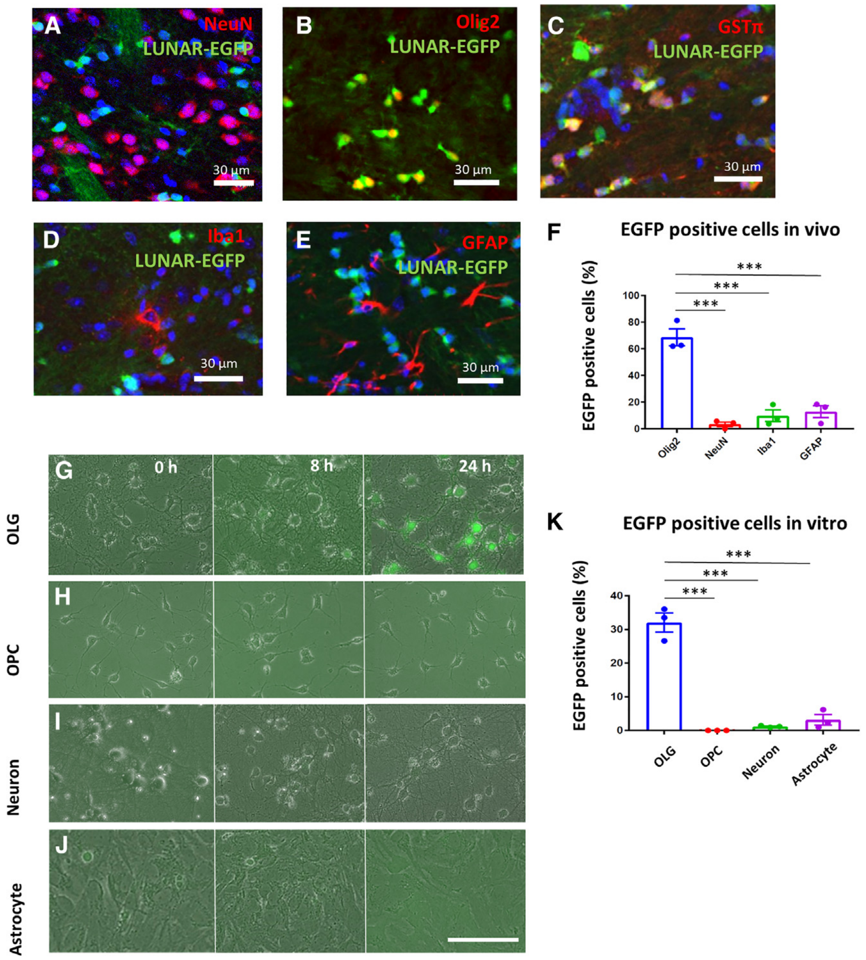


**Figure 2. LUNAR transfers EGFP mRNA in oligodendrocytes with high efficiency and specificity**

(A–E) EGFP-expressing cells colocalized with Olig2 and GSTπ. NeuN, Olig2, GSTπ, Iba1, GFAP (red), and EGFP (green) after 24-h treatment with LUNAR-*EGFP* mRNA. (F) The percentage of $EGFP^+$ cells colocalized with Olig2, NeuN, Iba1, and GFAP. Data are represented as mean ± SEM ($n = 3$). (G–J) The expression of EGFP (green) with LUNAR in rat primary culture, oligodendrocytes (OLG), oligodendrocyte precursor cells (OPC), neurons, and astrocytes after 0, 8, and 24 h. (K) The percentage of $EGFP^+$ cells in rat primary cultures after 24 h. Data are represented as mean ± SEM ($n = 3$); Tukey's test; $^{***}p < 0.001$.

Moreover, more than 65% of Olig2 was seen to be colocalized with EGFP, which was significantly higher than the colocalization of NeuN, GFAP, or Iba1 expression (Figure 2F). Next, the oligodendrocyte selectivity of LUNAR was confirmed using primary cell cultures from brain tissues. Primary cultures of neurons, astrocytes, oligodendrocyte precursor cells (OPCs), and oligodendrocytes were prepared from the cortex of Sprague-Dawley rats and treated with LUNAR-*EGFP* mRNA. Time-lapse imaging revealed that EGFP transferred with LUNAR-*EGFP* mRNA was gradually expressed only in oligodendrocytes, not in neurons (Videos S1 and S2). More than 30% of oligodendrocytes were seen to strongly express EGFP 24 h after treatment with LUNAR-*EGFP* mRNA (Figures 2G and 2K). Other cells showed a negligible expression of EGFP, thus indicating a significant difference from the EGFP expression in oligodendrocytes (Figures 1H–1K).

## LUNAR uptake into oligodendrocytes occurs via LDLR and ApoE receptor-2

ApoE protein and low-density lipoprotein receptor (LDLR) have been reported to be associated with liposome uptake.[16] First, we examined the association of LDLR, very LDLR (VLDLR), and apoprotein E receptor 2 (ApoER2) with LUNAR uptake in the mouse brain. LUNAR-*EGFP* mRNA was injected into the unilateral striatum of the brains of C57BL/6J mice that were sacrificed 24 h later for the histological analysis. EGFP and LDLR were seen to be colocalized with $Olig2^+$ cells, while ApoER2 or VLDLR did not show any such colocalization (Figures 3A–3L). An *in vitro* protein expression analysis of the primary culture demonstrated significant expression of *LDLR* and *VLDLR* mRNA in oligodendrocytes compared to that in other cell types (Figure 3M). Next, we analyzed the key molecule involved in the uptake of LUNAR using differentiated MO3.13 cells, a human oligodendrocytic cell line. *LDLR* knockdown significantly suppressed LUNAR uptake in differentiated MO3.13, while the knockdown of *VLDLR* or *ApoER2* did not have any effect on it (Figures 4A–4C). Moreover, cellular uptake of LUNAR did not occur in the absence of ApoE (Figure 4D). Therefore, ApoE proteins were added into LUNAR mixture before the treatment, which markedly enhance the EGFP expression (Figure 4D). We further found that cellular uptake of LUNAR was increased in the presence of fetal bovine serum (FBS), which contains various lipoproteins such as ApoE (Figure 4D). These data indicate that the uptake of LUNAR occurs via LDLR in the presence of ApoE.

## Twitcher mice rescued by LUNAR-*hGALC* mRNA

As mRNA can be delivered into oligodendrocytes with high efficiency and specificity by means of LUNAR, we evaluated the therapeutic effect of the LUNAR-*hGALC* mRNA in the treatment of Krabbe disease, which is associated with abnormalities in oligodendrocytes. The injection of LUNAR-*hGALC* mRNA into the unilateral striatum of adult mouse resulted in GALC proteins being expressed in oligodendrocytes compared with non-treated mice (Figures 5A–5F). LUNAR-*hGALC* mRNA was injected into the unilateral striatum of the Krabbe disease model mice (a GALC-deficient mouse, $Galc^{-/-}$, twitcher mice) at

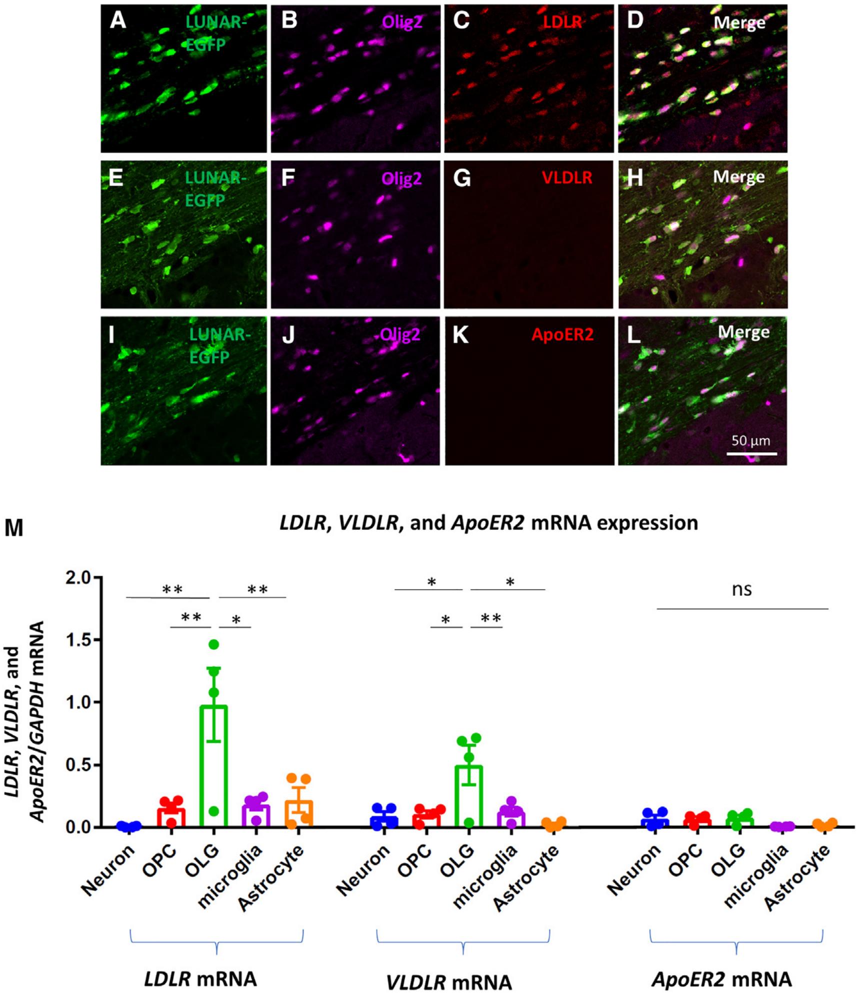


**Figure 3. LUNAR-*EGFP* mRNA is delivered into cells via LDLR**

(A–L) Double immunofluorescence staining of Olig2 (green) and LDLR, VLDLR, and ApoER2 (red) in the mouse brain after 24-h treatment with LUNAR-*EGFP* mRNA. (M) *LDLR*, *VLDLR*, and *ApoER2* mRNA expression in neurons, OPCs, oligodendrocytes, microglia, and astrocytes. Significantly increased *LDLR* and *VLDLR* mRNA levels seen in oligodendrocytes compared to those in neurons, OPCs, microglia, and astrocytes. Data are represented as mean ± SEM ($n$ = 4); Tukey's test; $^{*}p < 0.05$; $^{**}p < 0.01$; ns, not significant.

postnatal day 1 (P1) (Figure 5G). This treatment resulted in a significant increase in the Olig2$^{+}$ area on the injected side compared to that on the control side in twitcher mice at P35 (Figures 5H–5K). There was no significant difference in the Olig2$^{+}$ area between the injected side in twitcher mice and wild-type (WT) mice (Figures 5H–5K). Moreover, we injected LUNAR-*hGALC* mRNA into the bilateral striatum of twitcher mice at P1. The injected twitcher mice presented a significant decrease in the severity score compared to that of the control at P30 and P35, while exhibiting no significant difference in body weight (Figure 5L; Videos S3 and S4). LUNAR-*hGALC* mRNA injection extended the lifespan of twitcher mice up to 48 days, whereas that of the control mice was up to 42 days, thus showing a significant difference between the two groups (Figure 5M).

### Glycolipid analysis of twitcher and WT mice

GALC hydrolyzes galactosylceramide (GalCer) to galactose and ceramide and galactosylsphingosine (GalSph, psychosine) to galactose and sphingosine. Glycolipid profile analyses of WT, non-treated twitcher mice, and treated twitcher mice were performed at P14. Levels of GalCer decreased in non-treated twitcher mice and significantly increased in treated twitcher mice compared to those in WT mice (Figure 5N). However, GalSph levels were elevated in both treated and non-treated twitcher mice compared to those in WT mice and did not differ significantly between the two groups (Figure 5N).

## DISCUSSION

The field of DDSs involving the use of LNPs has made remarkable progress. However, technologies having high specificity in delivering drugs to particular cell types have not been developed. Although previous studies investigated the efficiency of luciferase expression on an organ-by-organ basis, there are few reports that present detailed histological analyses, especially of the brain. In this study, we conducted a histological analysis of cell types transduced by LUNAR *EGFP* mRNA in the brain and found LUNAR to be a cell-type-specific nanocarrier having high efficiency of transduction and specificity to oligodendrocytes.

In general, LNPs containing cholesterol tend to be taken up by the liver. Previous studies have reported that the uptake of LNPs by the liver occurs via LDLR by means of their binding to ApoE.[16] LUNAR is also a cholesterol-containing LNP that is designed and engineered for efficient uptake by the liver. A clinical trial to treat liver diseases, such as ornithine transcarbamylase deficiency, using LUNAR is under way. Considering the importance of LDLR in the process of LNP uptake, we examined apoprotein receptors in the brain and found that oligodendrocytes have a high expression of LDLR. Furthermore, *in vitro* cell line studies showed the uptake of LUNAR to occur via LDLR in the presence of ApoE. These results indicate that oligodendrocytes, which specifically express LDLR in the brain, can efficiently take up LUNAR bound to ApoE by recognizing it. In the present study, we thus combined cell-specific nanocarriers with therapeutic mRNA and succeeded in introducing mRNA into target cells.

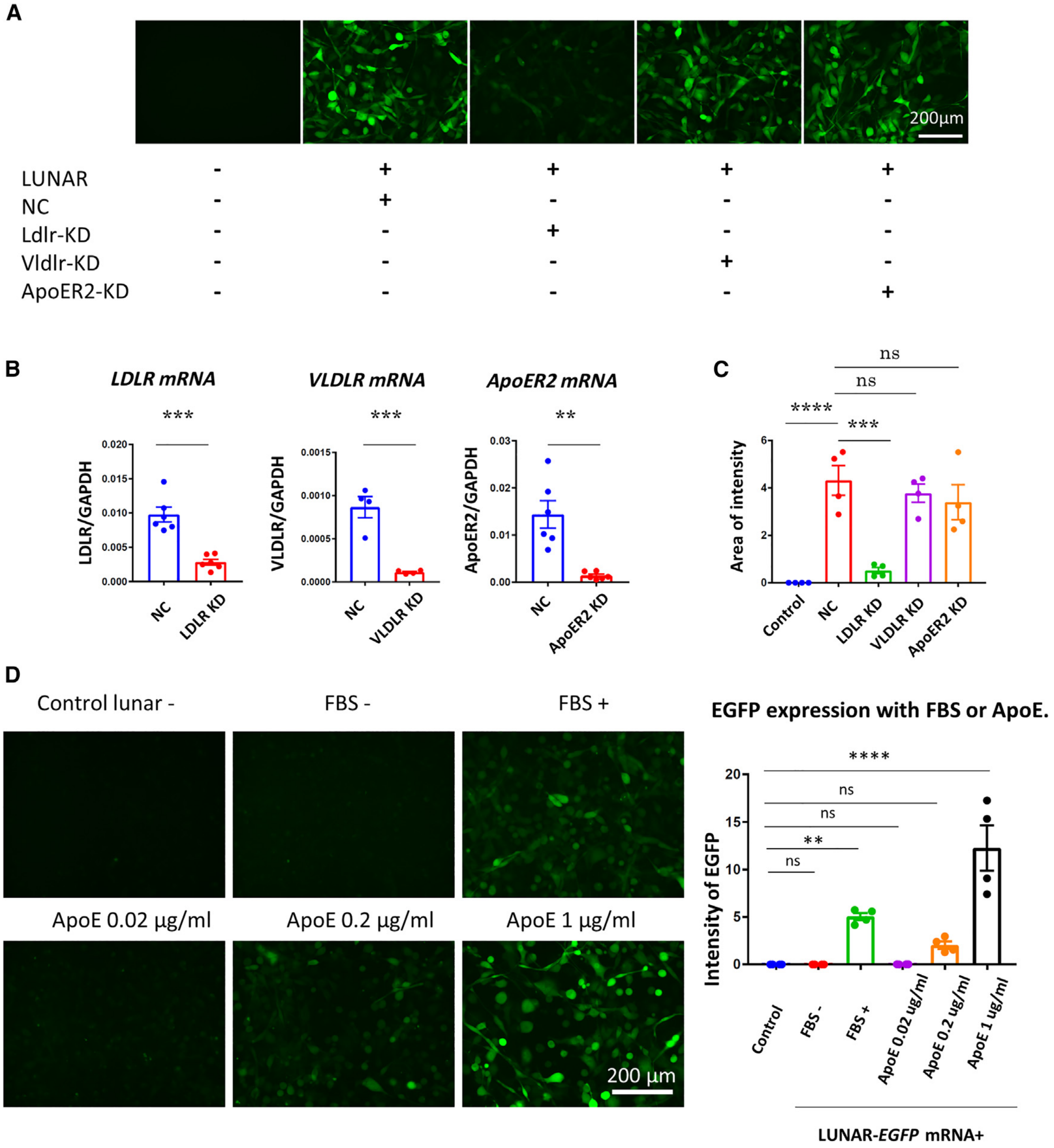


**Figure 4. LUNAR uptake occurring in the presence of FBS or ApoE**

(A) Representative images of differentiated MO3.13 cells expressing EGFP with LUNAR showing EGFP expression suppressed by *LDLR* knockdown (KD). LUNAR, LUNAR-*EGFP* mRNA; NC, negative control siRNA. (B) Expression of LDLR, VLDLR, and ApoER2 mRNA significantly reduced by *LDLR, VLDLR*, and *ApoER2* siRNA in MO3.13 cells. Data are represented as mean ± SEM ($n = 6$); t test. $^{**}p < 0.01$; $^{***}p < 0.001$. (C) LUNAR uptake significantly reduced by *LDLR* knockdown. Data are represented as mean ± SEM ($n = 4$); Tukey's test; $^{**}p < 0.01$; $^{***}p < 0.001$; $^{****}p < 0.0001$. (D) Figures depict LUNAR uptake with and without FBS/ApoE. EGFP expression was significantly increased in the presence of FBS or ApoE. Data are represented as mean ± SEM ($n = 4$); Dunnett's test; $^{**}p < 0.01$; $^{****}p < 0.0001$.

Moreover, we demonstrated that the characteristic of oligodendrocyte-specific uptake of LUNAR could be utilized to make it a useful DDS for mRNA-based treatment of Krabbe disease. We used LUNAR to deliver *GALC*-mRNA specifically into oligodendrocytes in twitcher mice and observed improvement in motor function and survival in the treated mice. GalCer is enriched in myelin and is important for stabilizing myelin.[17] GalCer is one of the precursors of cytotoxic GalSph production.[18,19] The accumulation of cytotoxic GalSph in the brain has been proposed to play a central role in the pathological mechanism of Krabbe disease.[20] It has been reported that GalSph levels increased, whereas GalCer levels decreased in the brains from patients with Krabbe disease. The reduction in GalCer levels might be due to

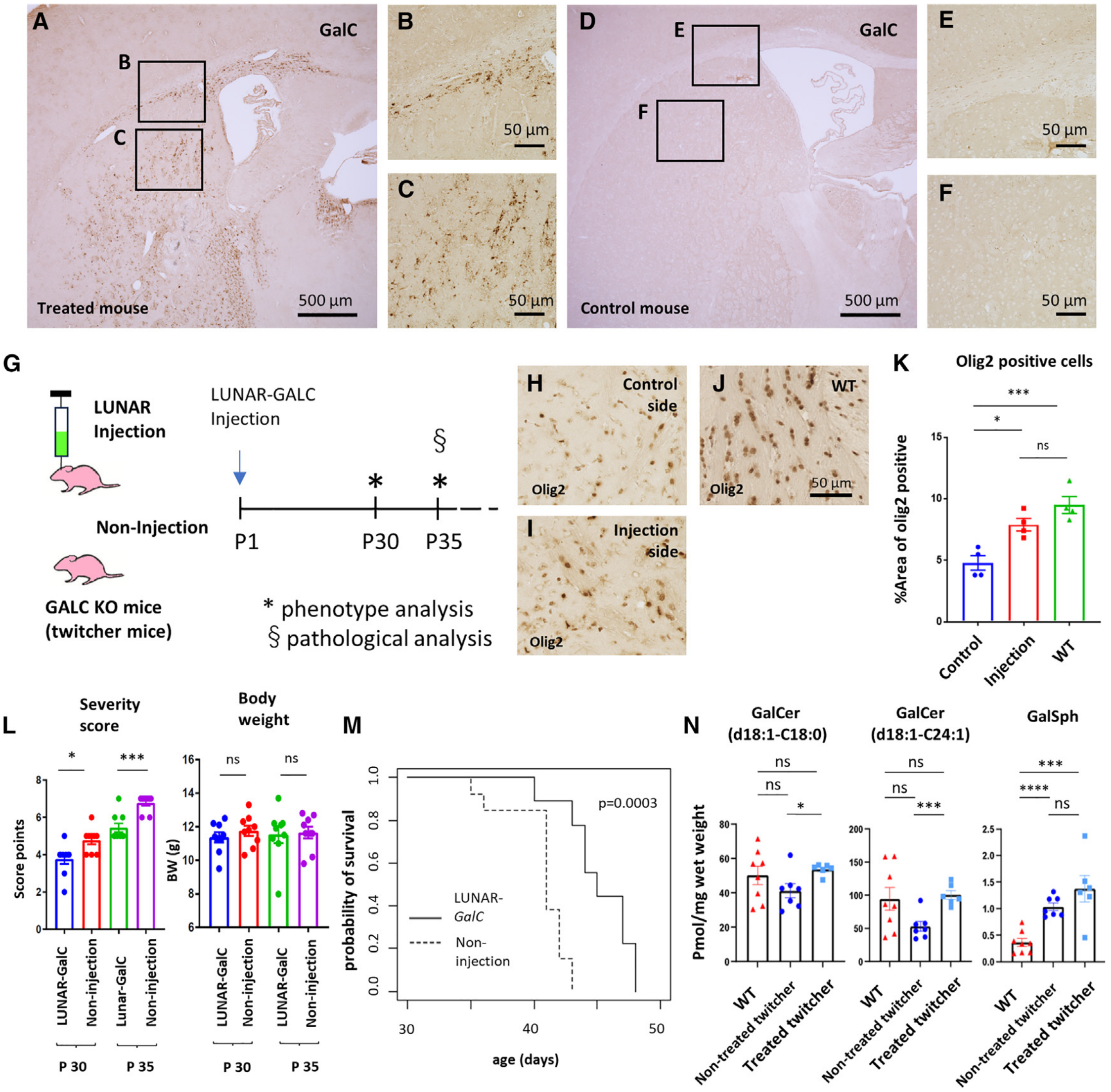


**Figure 5. GALC expression induced by LUNAR ameliorates the phenotype of twitcher mice**

(A–C) Immunohistochemistry of GALC in mouse brain injected with LUNAR-*GALC* mRNA. (D–F) Immunohistochemistry of GALC in control mouse brain. (G) Schematic representation of the experimental design. (H and I) Representative images of Olig2$^+$ cells on the control and injection sides in twitcher mouse at P35. (J) Representative images of Olig2$^+$ cells in WT mouse at P35. (K) Percentage area of Olig2$^+$ cells on the control and injection sides in twitcher mice and in WT mice. Data are represented as mean ± SEM ($n$ = 4); Tukey's test; *$p < 0.05$; ***$p < 0.001$. (L) The severity score and body weight of twitcher mice at P30 and P35. Control (non-injection) ($n$ = 13) and LUNAR-*hGALC* mRNA-treated mice ($n$ = 9). Data are represented as mean ± SEM. (M) The Kaplan-Meier survival curves in twitcher mice with or without LUNAR-*hGALC* mRNA injections. Survival of untreated (non-injection) ($n$ = 13) and LUNAR-*hGALC* mRNA-treated mice ($n$ = 9). The overall difference in the survival among non-injection and LUNAR-*hGALC* mRNA-treated mice was highly significant ($p$ = 0.0003 by the log rank test). (N) Analysis of GalCer (d18:1-C18:0), GalCer (d18:1-C24:1), GalSph of WT mice ($n$ = 7), non-treated twitcher mice ($n$ = 7), and treated twitcher mice ($n$ = 6). Data are represented as mean ± SEM; Tukey's test; *$p < 0.05$; ***$p < 0.001$; and ****$p < 0.0001$.

demyelination.[21] Results of the present study do not show a decrease in GalSph levels but show an increase in GalCer levels in treated twitcher mice. In the CNS, GalCer is synthesized in oligodendrocytes[22]; hence, the observed increase in GalCer levels in treated twitcher mice is interpreted as being due to the recovery of oligodendrocytes. In addition, the observed increase in GalSph levels is considered to be due to the conversion of excessive GalCer into GalSph.

Our results thus show LUNAR to be capable of delivering mRNAs to oligodendrocytes with high efficiency and specificity, where efficient gene transfer is usually difficult. LUNAR can therefore be considered a novel mRNA therapeutic tool for diseases affecting oligodendrocytes, such as Krabbe disease, multiple system atrophy, Alexander disease, PMD, adrenoleukodystrophy, and other leukodystrophies. Moreover, LUNAR has the advantage of being capable of mRNA

transduction without involving the use of a viral vector and hence does not require consideration of virus-induced inflammation or infectivity. The development of mRNA-based vaccines against COVID-19 has led to increased attention toward mRNA therapy because of concerns regarding a higher risk of thrombosis associated with viral vector-based vaccines compared to the risk posed by mRNA-based vaccines.[23] Despite the high cell directivity of LUNAR, its medicinal development is still in its infancy and has yet to be optimized as well as AAV vector methodologies have been. In fact, it has been reported that the combination of AAV vectors and stem cell therapy enhances the efficacy.[24] The combination of LUNAR and stem cell therapy should be considered in the future. Further improvement in DDSs is needed to establish highly efficient transduction of mRNA to the brain with minimal invasion via intravenous administration, even though a high degree of invasiveness can be justified to be acceptable in the case of lethal diseases such as Krabbe disease. In the present study, we observed that EGFP expression by LUNAR *EGFP* mRNA diminished after 14 days (Figure 1G). In the future, it might be necessary to increase dosing or use a self-replicating RNA to prolong protein expression.

A variety of cell-type-specific nanocarriers for various neurological diseases can be developed if the transduction of mRNA to various other cells besides oligodendrocytes, such as neurons, microglia, and astrocytes, could be performed with high efficiency and specificity. This new concept of LNP-based nanocarriers targeting specific cells has potential applications in the treatment of many neurological diseases owing to its ability to specifically control various cell types in the brain, thus creating a need for further research and development of clinical applications.

## MATERIALS AND METHODS

### mRNA synthesis

Codon optimization of human GALC (*hGALC*, NCBI Reference Sequence: NP_000144.2) and selection of 5′ UTR and 3′ UTR sequences were performed using proprietary algorithms developed at Arcturus Therapeutics (San Diego, CA). The human codon-optimized *hGALC* was cloned between the 5′ UTR and 3′ UTR in a plasmid that was engineered for *in vitro* transcription (IVT). The sequence of the cloned portion in the plasmid was verified by DNA sequencing. The plasmid was linearized immediately after the poly(A) stretch located downstream of the 3′ UTR and used as a template for IVT reaction with T7 RNA polymerase.

The IVT reactions were performed as previously described,[10] but with 100% substitution of Uridine-5'-triphosphate (UTP) with N1-methyl-pseudouridine (N1mΨ). The RNA quality and integrity were verified by 0.8%–1.2% non-denaturing agarose gel electrophoresis as well as Fragment Analyzer (Advanced Analytical). The purified RNAs were stored in RNase-free water at −80°C until further use.

### Preparation of liposomes and mRNA

We used the proprietary LUNAR lipid delivery technology platform supplied by Arcturus Therapeutics. LNPs were prepared by using a NanoAssemblr microfluidic device to mix appropriate volumes of lipids in ethanol with an aqueous phase containing *EGFP* or *hGALC* mRNA and then subjecting the mixture to downstream processing. For the encapsulation of mRNA by the LNPs, the desired amount of RNA was dissolved in 5 mmol/L citric acid buffer (pH 3.5). Lipids having the desired molar concentrations were dissolved in ethanol. The mole concentrations for the constituent lipids were 0.16 mM ATX (a class of proprietary ionizable amino lipids), 0.02 mM 1,2-dioctadecanoyl-*sn*-glycerol-3-phosphocholine (Avanti Polar Lipids, Alabaster, AL), 0.12 mM cholesterol (Avanti Polar Lipids), and 0.05 mM 1,2-dimyristoyl-*sn*-glycerol, methoxypolyethylene glycol, polyethylene glycol chain molecular weight: 2,000 (NOF America, White Plains, NY).[25–28] At a 1:3 flow ratio of ethanol:aqueous phases, the solutions were combined in a microfluidic device (Precision NanoSystems, Vancouver, BC, Canada). The total combined flow rate was 12 mL/min per microfluidic chip. LNPs thus formed were purified by dialysis against phosphate buffer overnight using 100 kDa Spectra/Por Float-A-Lyzer ready-to-use dialysis device (Repligen, Waltham, MA) followed by concentration using Amicon Ultra-15 centrifugal filters (Merck Millipore, Burlington, MA). The particle size was determined by dynamic light scattering (ZEN3600, Malvern Instruments, Malvern, UK). The encapsulation efficiency was calculated by determining the unencapsulated mRNA content by measuring the fluorescence upon the addition of RiboGreen (Molecular Probes, Eugene, OR) to the LNP slurry (Fi) and comparing this value to the total mRNA content obtained upon lysis of the LNPs with 1% Triton X-100 (Ft), where percentage encapsulation = (Ft − Fi)/Ft × 100.

### Animals

C57BL/6J male mice at 2 months of age (purchased from Japan SLC, Shizuoka, Japan) ($n = 60$) and Sprague-Dawley rats ($n = 15$) were used for the experiments. The mice were maintained at 25°C with 55% humidity on a 12-h light-dark cycle and given free access to food and drinking water. All experimental procedures used in this study followed Japanese national guidelines on animal experimentation. Ethical approval and permission were obtained from The Animal Experimentation Committee of Kyoto University (MedKyo, 19219). Twitcher mice were purchased from The Jackson Laboratory (Bar Harbor, ME). The genotypes were determined by analyzing DNA from the mouse tail that was extracted using Hypercool Primer & Probe (Nihon Gene Laboratory, Miyagi, Japan). Primers and probes used were as follows: forward primer, CTTTTAACGTTGTCT CATTCAC; reverse primer, CTAGATGGCCCACTGTC; WT probe, FAM-aaTACCAGccTGGTTGaGTA-BHQ; and mutant probe, HEX-aaTaTcAGccTGGTTGaGTaA-BHQ. The signal was detected using Luna Universal Probe qPCR Master Mix (M3004, New England Biolabs, Ipswich, MA) with LightCycler 480 (Roche, Basel, Switzerland).

### Surgery

The adult mice were anesthetized with isoflurane for injections of the LNPs into the dorsal striatum. Unilateral stereotaxic injections (2 μL) of LUNAR carrying *EGFP*, *hGALC*, or *h*mRNA were administered into the dorsal striatum (coordinates: 2.0 mm relative to

bregma; 0.2 mm from the midline; 2.5 mm beneath the skull surface) using a 33G syringe. For neonatal mice, injections of 0.5 μL LUNAR-*hGALC* mRNA were administered into the bilateral striatum using a 30G syringe.

### Histological analysis of mouse brain tissues

LUNAR-injected mice were sacrificed at the indicated time points. Following perfusion with 4% (w/v) paraformaldehyde in PBS, the brains were removed and subjected to overnight immersion in 4% (w/v) paraformaldehyde in PBS at 4°C. Tissues were sectioned using a vibratome (Neo-LinearSlicer, DOSAKA, Kyoto, Japan) or embedded in paraffin for sectioning. Paraffin sections of 8 μm thickness were prepared using an HM 325 rotary microtome (Microm, Thermo Fisher Scientific, Waltham, MA).

### Immunohistochemistry

The following primary antibodies were used for immunohistochemical analysis: anti-NeuN (Millipore, ABN78, 1:400), anti-Iba1 (Wako, Osaka, Japan, 019–19741, 1:200), anti-GFAP (Invitrogen, Carlsbad, CA, 13–0300, 1:200), anti-GSTπ (MBL International, Schaumburg, IL, 312, 1:500), anti-Olig2 (Millipore, ab9610, 1:300), anti-GFP (Abcam, Cambridge, UK, ab13970, 1:300), and anti-GALC (Abcam, ab232972, 1:1,000). The sections were incubated overnight at 4°C with primary antibodies in skim milk or BSA and then processed for visualization. Histofine Simple Stain (Nichirei Biosciences, Tokyo, Japan) was used as a secondary antibody for diaminobenzidine staining, and Alexa Fluor 488- or 594-conjugated antibodies (Thermo Fisher Scientific) were used for immunofluorescence. Sections were examined using a BZ-X710 fluorescence microscope (KEYENCE, Osaka, Japan) and an FV-1000 confocal laser scanning microscope (Olympus, Tokyo, Japan).

### Cultures of primary oligodendrocyte lineage cells and other glial cells

Primary rat OPCs were prepared according to methods described in previous publications.[29–31] Cerebral cortices from 1- to 2-day-old Sprague-Dawley rats (Shimizu Laboratory Supplies, Kyoto, Japan) were dissected, minced, and digested. Dissociated cells were plated in 75 $cm^2$ poly-D-lysine-coated flasks and maintained in DMEM (Thermo Fisher) containing 20% heat-inactivated FBS and 1% penicillin/streptomycin. When the cells became confluent (∼10 days), the flasks were shaken for 1 h on an orbital shaker (220 rpm) at 37°C to remove microglia. The flasks were then replaced with fresh medium and shaken overnight (∼20 h). After incubation for 1 h at 37°C on non-coated cell culture dishes, the non-adherent cells (OPCs) were replated at a density of 20,000 cells/$cm^2$ in Neurobasal Medium (Thermo Fisher) containing 2 mM glutamine, 1% penicillin/streptomycin, 10 ng/mL platelet-derived growth factor-AA, 10 ng/mL fibroblast growth factor-2, and 2% B27 supplement onto poly-DL-ornithine-coated plates. The culture medium was replaced with DMEM containing 1% penicillin/streptomycin, 10 ng/mL ciliary neurotrophic factor, 15 nM T3, and 2% B27 supplement for the OPCs to differentiate into mature oligodendrocytes. The OPCs and oligodendrocytes were treated with LUNAR-*EGFP* mRNA (0.2 μL/well), and the EGFP expression of these cells was observed using a BZ-X710 fluorescence microscope after 0, 8, and 24 h.

### Primary neuronal cell cultures

Cortical neuronal cultures were prepared from 17-day-old Sprague-Dawley rat embryos according to a method described in previous publications.[29–31] Cortices were first dissected and dissociated. The cells were then plated into dishes coated with poly-D-lysine in DMEM containing 5% FBS and 1% penicillin/streptomycin at a density of 200,000 cells/$cm^2$. At 24 h after seeding, the medium was changed to Neurobasal Medium (Thermo Fisher) containing 0.5 mM L-glutamine, 1% penicillin/streptomycin, and 2% B27 supplement. Cultured neurons were used for the experiments 14 days after seeding.

### Cell line culture

Cells from the human oligodendrocyte cell line MO3.13 (RRID: CVCL_D357) were cultured according to a method described in a previous publication.[32] MO3.13 cells were cultured for 48–72 h in high glucose DMEM (Wako, 043–30085) with 10% FBS (Gibco, Grand Island, NY) and a mixture of penicillin 100 U/mL and streptomycin 100 μg/mL (Nacalai Tesque, Kyoto, Japan, 26253-84) in a humidified incubator with 5% $CO_2$ at 37°C. For cell maturation studies, 16–24 h after seeding cells, the culture medium was replaced with DMEM containing FBS 0%, penicillin 100 U/mL, streptomycin 100 μg/mL, and 100 nM phorbol 12-myristate 13-acetate (Sigma-Aldrich, St. Louis, MO, P1585). Cells were allowed to mature for 3–7 days with a change in medium on alternate days.

### siRNA treatments

Seeded cells were incubated for 24 h and transfected with small interfering RNA (siRNA) using Lipofectamine RNAiMAX reagent (Thermo Fisher Scientific), according to the manufacturer's instructions. After 24 h of incubation, the medium was replaced with fresh medium and the cells were treated with LUNAR *EGFP* mRNA for another 24 h. We used ON-TARGET*plus* siRNAs to knock down LDLR, VLDLR, and ApoER2 (Horizon Discoveries, Cambridge, UK, L-011073, L-003721, and L-011802, respectively). *Silencer* Select Negative Control 2 siRNA (Thermo Fisher Scientific, 4390846) was used as the control siRNA.

### Quantitative real-time PCR

Total RNA was isolated using a QIAGEN RNeasy Mini Kit (Qiagen, Venlo, Nederland, 74106), reverse transcribed into cDNA (PrimeScript RT Master Mix, Takara, Shiga, Japan, RR036), amplified, and quantified by SYBR Green (Thermo Fisher, K0222) detection. All quantitative real-time PCRs were run with LightCycler 480 (Roche). Relative mRNA expression was normalized with the glyceraldehyde 3-phosphate dehydrogenase gene. All primers are listed in Table S1.

### Neuropathological analysis

To assess the distribution and efficiency of LUNAR-*EGFP* mRNA *in vivo,* two locations in each brain ($n$ = 3) region injected with

**Table 1. Analytical conditions used for the analysis by MRM methods**

| Analyte | Precursor ion (Q1) $[M + H]^+$ | Product ion (Q3) long-chain base-related ions | Collision energy, eV |
|---|---|---|---|
| GlcSph and GalSph | 462.3 | 282.1 | 27 |
| GlcSph-*d*5 | 467.3 | 287.1 | 27 |
| GlcCer (d18:1-C12:0) | 644.3 | 264.2 | 43 |
| GlcCer and GalCer (d18:1-C18:0) | 728.6 | 264.2 | 48 |
| GlcCer and GalCer (d18:1-C24:1) | 810.7 | 264.2 | 55.5 |

GalCer, galactosylceramide; GalSph, galactosylsphingosine; GlcCer, β-D-glucopyranosyl-(1→1)-*N*-lauroyl-D-*erythro*-sphingosine; GlcSph-*d*5, β-D-glucopyranosyl-(1→1)-D-*erythro*-sphingosine-*d*5; MRM, multiple reaction monitoring.

LUNAR were screened at 20× magnification using a BZ-X710 fluorescence microscope (KEYENCE). Cells positive for cell-specific markers (Olig2, NeuN, Iba1, and GFAP) and EGFP-cell marker double-positive cells were counted using ImageJ software. The $EGFP^+$ ratio *in vivo* was calculated as (number of double-positive cells)/(number of cell marker-positive cells). For *in vitro* experiments, we screened four places in each well ($n = 3$) of neurons, oligodendrocytes, OPCs, and GFAP at 0, 8, and 24 h after treatment with LUNAR-*EGFP* mRNA. Total and $EGFP^+$ cells were counted. The *in vitro* $EGFP^+$ ratio was calculated as (number of LUNAR-$EGFP^+$ cells)/(total number of cells). Tukey's test was used to compare the statistical significance.

### Assessment of clinical symptoms

Body weight and neurological symptoms of these mice at P30 and P35 were assessed using the following system for scoring the severity of the twitching, as was reported in a previous publication[33]: frequency: 1, rare; 2, intermittent; 3, constant; and severity: 1, asymptomatic; 2, mild; 3, mild moderate; 4, moderate; 5, severe. The final score is the sum of the two parameters.

### Materials for glycolipid analysis

β-D-Glucopyranosyl-(1→1)-*N*-lauroyl-D-*erythro*-sphingosine (GlcCer [d18:1-C12:0]) and β-D-glucopyranosyl-(1→1)-D-*erythro*-sphingosine-*d*5 (GlcSph-*d*5) were purchased from Avanti Polar Lipids. Liquid chromatography (LC)-electrospray ionization tandem mass spectrometry (ESI-MS/MS) was performed using high-performance LC-grade acetonitrile, methanol, and distilled water purchased from Kanto Chemical (Tokyo, Japan), chloroform and formic acid purchased from FUJIFILM Wako, and ammonium formate purchased from Sigma-Aldrich.

### Lipid extraction for analysis of the glycolipid profile

Frozen tissue (half of the brain, approximately 1 g) was lyophilized. The lyophilized tissues comprising half of the brain (approximately 25 mg) and the frozen striatum (approximately 40 mg) were homogenized, and total lipids were extracted with a chloroform:methanol (C:M) (2:1, v/v, 3–5 mL) mixture added to 5 pmol/mg lyophilized tissue or to 1 pmol/mg frozen tissue of GlcCer (d18:1-C12:0), and GlcSph-*d*5 served as an internal standard. The extracts were dried under a flow of $N_2$ gas and hydrolyzed for 2 h at room temperature in C:M (2:1, v/v, 2 mL) containing 0.1 M KOH. The reaction mixture was neutralized with 7.5 μL glacial acetic acid. The neutralized reaction mixture was subjected to Folch's partition, and the lower phase was dried under a flow of $N_2$ gas. The resulting lipid films were suspended in C:M (2:1, v/v) at a concentration of 20 μg of lyophilized tissue/μL or 100 μg of frozen tissue/μL, and aliquots were subjected to LC-ESI-MS/MS.

### LC-ESI-MS/MS for glycolipid analysis

LC-ESI-MS/MS was performed on an LC system (Nexera X2, Shimadzu, Kyoto, Japan) attached to a triple-quadrupole linear ion trap mass spectrometer (QTRAP4500; SCIEX, Tokyo, Japan). The LC-ESI-MS/MS datasets were analyzed using MultiQuant (version 2.1) and Analyst (SCIEX) software programs. Target lipids were monitored in multiple reaction monitoring (MRM) mode using specific precursor-product ion pairs, as described in Table 1. GalCer and GalSph were analyzed by hydrophilic interaction chromatography (HILIC)-ESI-MS/MS. HILIC enables the separation of GlcCer and GalCer and GlcSph and GalSph.[34] The lipid extracts dissolved in C:M (2:1, v/v) were diluted 10-fold with mobile phase A (acetonitrile:methanol:formic acid, 97:2:1 [v/v/v], with 5 mM ammonium formate), and aliquots (10 μL) were applied to an Atlantis silica HILIC column (2.1 mm inner diameter × 150 mm, particle size, 3 μm; Waters, Milford, MA) maintained at 40°C. HILIC-ESI-MS/MS analysis for GalCer was performed according to a previously established method,[35,36] with minor modifications. The mass spectrometer was set to positive ion mode (ion spray voltage, 5,500 V; curtain gas pressure, 30 psi; nebulizer gas pressure, 50 psi; heating gas pressure, 30 psi; temperature, 100°C) using MRM detection for targeted analysis. HILIC-ESI-MS/MS analysis for GalSph was performed according to a previously established method,[36,37] with minor modifications. For this process, the mass spectrometer was set to positive ion mode (ion spray voltage, 5,500 V; curtain gas pressure, 30 psi; nebulizer gas pressure, 70 psi; heating gas pressure, 80 psi; temperature, 700°C) using MRM detection for targeted analysis. The ionization efficiencies of GlcCer and GalCer and GlcSph and GalSph were similar under the implemented conditions. Peak areas were integrated and quantified relative to the associated internal standard.

### Statistical analysis

The experiments were performed three to four times independently. All quantitative data were analyzed using GraphPad Prism software version 7. Multiple comparisons were evaluated by a one-way ANOVA followed by the Tukey-Kramer test or Dunnett's test. All values are represented as mean ± SEM. Statistical significance was defined as $p < 0.05$.

## DATA AND CODE AVAILABILITY

All data needed to evaluate the conclusions in the paper are present in the paper and supplemental information.

## ACKNOWLEDGMENTS

We thank Yasuko Matsuzawa for technical assistance, Josie Rivera Alfaro for her assistance with DNA preparation and mRNA synthesis by IVT, Alana Montoya and Kristen Kuakini for their assistance with mRNA purification and quality check, and Tim Luger for his assistance with mRNA purification. This study was supported by Arcturus Therapeutics Inc. (to S.M.) and RIKEN Pioneering Project (Glycolipidologue Initiative) (to Y.H.).

## AUTHOR CONTRIBUTIONS

M.S., K.T., R.T., and S.M. conceptualized the projects and aims, with input from P.C., K.T., and H.Y. A.I.L., H.H., and R.M. designed and prepared mRNA and LNPs. M.S., S.K., K.Y., and R.H. performed the cell culture, qPCR, and immunohistochemistry assays. H.A. and Y.H. performed the glycolipid analysis. M.S. and S.M. wrote the manuscript, with input from the authors.

## DECLARATION OF INTERESTS

K.T., A.I.L., H.H., R.M., and P.C. are employees of Arcturus Therapeutics Inc.

## SUPPLEMENTAL INFORMATION

Supplemental information can be found online at https://doi.org/10.1016/j.omtn.2024.102380.

## REFERENCES

1. Finkel, R.S., Mercuri, E., Darras, B.T., Connolly, A.M., Kuntz, N.L., Kirschner, J., Chiriboga, C.A., Saito, K., Servais, L., Tizzano, E., et al. (2017). Nusinersen versus Sham Control in Infantile-Onset Spinal Muscular Atrophy. N. Engl. J. Med. *377*, 1723–1732.
2. Mercuri, E., Darras, B.T., Chiriboga, C.A., Day, J.W., Campbell, C., Connolly, A.M., Iannaccone, S.T., Kirschner, J., Kuntz, N.L., Saito, K., et al. (2018). Nusinersen versus Sham Control in Later-Onset Spinal Muscular Atrophy. N. Engl. J. Med. *378*, 625–635.
3. Moser, H.W. (2006). Peripheral nerve involvement in Krabbe disease: a guide to therapy selection and evaluation. Neurology *67*, 201–202.
4. Engelen, M., Kemp, S., de Visser, M., van Geel, B.M., Wanders, R.J.A., Aubourg, P., and Poll-The, B.T. (2012). X-linked adrenoleukodystrophy (X-ALD): clinical presentation and guidelines for diagnosis, follow-up and management. Orphanet J. Rare Dis. *7*, 51.
5. von Jonquieres, G., Fröhlich, D., Klugmann, C.B., Wen, X., Harasta, A.E., Ramkumar, R., Spencer, Z.H.T., Housley, G.D., and Klugmann, M. (2016). Recombinant Human Myelin-Associated Glycoprotein Promoter Drives Selective AAV-Mediated Transgene Expression in Oligodendrocytes. Front. Mol. Neurosci. *9*, 13.
6. Powell, S.K., Khan, N., Parker, C.L., Samulski, R.J., Matsushima, G., Gray, S.J., and McCown, T.J. (2016). Characterization of a novel adeno-associated viral vector with preferential oligodendrocyte tropism. Gene Ther. *23*, 807–814.
7. Li, H., Okada, H., Suzuki, S., Sakai, K., Izumi, H., Matsushima, Y., Ichinohe, N., Goto, Y.I., Okada, T., and Inoue, K. (2019). Gene suppressing therapy for Pelizaeus-Merzbacher disease using artificial microRNA. JCI Insight *4*, e125052.
8. Georgiou, E., Sidiropoulou, K., Richter, J., Papaneophytou, C., Sargiannidou, I., Kagiava, A., von Jonquieres, G., Christodoulou, C., Klugmann, M., and Kleopa, K.A. (2017). Gene therapy targeting oligodendrocytes provides therapeutic benefit in a leukodystrophy model. Brain *140*, 599–616.
9. Tamaru, M., Akita, H., Nakatani, T., Kajimoto, K., Sato, Y., Hatakeyama, H., and Harashima, H. (2014). Application of apolipoprotein E-modified liposomal nanoparticles as a carrier for delivering DNA and nucleic acid in the brain. Int. J. Nanomedicine *9*, 4267–4276.
10. Ramaswamy, S., Tonnu, N., Tachikawa, K., Limphong, P., Vega, J.B., Karmali, P.P., Chivukula, P., and Verma, I.M. (2017). Systemic delivery of factor IX messenger RNA for protein replacement therapy. Proc. Natl. Acad. Sci. USA *114*, E1941–E1950.
11. Yanagi, T., Tachikawa, K., Wilkie-Grantham, R., Hishiki, A., Nagai, K., Toyonaga, E., Chivukula, P., and Matsuzawa, S.I. (2016). Lipid Nanoparticle-mediated siRNA Transfer Against PCTAIRE1/PCTK1/Cdk16 Inhibits In Vivo Cancer Growth. Mol. Ther. Nucleic Acids *5*, e327.
12. Suzuki, K., and Suzuki, Y. (1970). Globoid cell leucodystrophy (Krabbe's disease): deficiency of galactocerebroside beta-galactosidase. Proc. Natl. Acad. Sci. USA *66*, 302–309.
13. Nagara, H., Ogawa, H., Sato, Y., Kobayashi, T., and Suzuki, K. (1986). The twitcher mouse: degeneration of oligodendrocytes in vitro. Brain Res. *391*, 79–84.
14. Tanaka, K., and Webster, H.D. (1993). Effects of psychosine (galactosylsphingosine) on the survival and the fine structure of cultured Schwann cells. J. Neuropathol. Exp. Neurol. *52*, 490–498.
15. Taniike, M., Mohri, I., Eguchi, N., Irikura, D., Urade, Y., Okada, S., and Suzuki, K. (1999). An apoptotic depletion of oligodendrocytes in the twitcher, a murine model of globoid cell leukodystrophy. J. Neuropathol. Exp. Neurol. *58*, 644–653.
16. Akita, H., Noguchi, Y., Hatakeyama, H., Sato, Y., Tange, K., Nakai, Y., and Harashima, H. (2015). Molecular Tuning of a Vitamin E-Scaffold pH-Sensitive and Reductive Cleavable Lipid-like Material for Accelerated in Vivo Hepatic siRNA Delivery. ACS Biomater. Sci. Eng. *1*, 834–844.
17. Baumann, N.A., Harpin, M.L., and Bourré, J.M. (1970). Long chain fatty acid formation: key step in myelination studied in mutant mice. Nature *227*, 960–961.
18. Li, Y., Xu, Y., Benitez, B.A., Nagree, M.S., Dearborn, J.T., Jiang, X., Guzman, M.A., Woloszynek, J.C., Giaramita, A., Yip, B.K., et al. (2019). Genetic ablation of acid ceramidase in Krabbe disease confirms the psychosine hypothesis and identifies a new therapeutic target. Proc. Natl. Acad. Sci. USA *116*, 20097–20103.
19. Yaghootfam, C., Gehrig, B., Sylvester, M., Gieselmann, V., and Matzner, U. (2021). Deletion of fatty acid amide hydrolase reduces lyso-sulfatide levels but exacerbates metachromatic leukodystrophy in mice. J. Biol. Chem. *297*, 101064.
20. Igisu, H., and Suzuki, K. (1984). Progressive accumulation of toxic metabolite in a genetic leukodystrophy. Science (New York, N.Y.) *224*, 753–755.
21. Svennerholm, L., Vanier, M.T., and Månsson, J.E. (1980). Krabbe disease: a galactosylsphingosine (psychosine) lipidosis. J. Lipid Res. *21*, 53–64.
22. Schulte, S., and Stoffel, W. (1993). Ceramide UDPgalactosyltransferase from myelinating rat brain: purification, cloning, and expression. Proc. Natl. Acad. Sci. USA *90*, 10265–10269.
23. Rzymski, P., Perek, B., and Flisiak, R. (2021). Thrombotic Thrombocytopenia after COVID-19 Vaccination: In Search of the Underlying Mechanism. Vaccines *9*, 559.
24. Rafi, M.A., Luzi, P., and Wenger, D.A. (2020). Conditions for combining gene therapy with bone marrow transplantation in murine Krabbe disease. Bioimpacts *10*, 105–115.
25. Mui, B.L., Tam, Y.K., Jayaraman, M., Ansell, S.M., Du, X., Tam, Y.Y.C., Lin, P.J., Chen, S., Narayanannair, J.K., Rajeev, K.G., et al. (2013). Influence of Polyethylene Glycol Lipid Desorption Rates on Pharmacokinetics and Pharmacodynamics of siRNA Lipid Nanoparticles. Molecular therapy. Nucl. Acid. *2*, e139.
26. Akinc, A., Querbes, W., De, S., Qin, J., Frank-Kamenetsky, M., Jayaprakash, K.N., Jayaraman, M., Rajeev, K.G., Cantley, W.L., Dorkin, J.R., et al. (2010). Targeted delivery of RNAi therapeutics with endogenous and exogenous ligand-based mechanisms. Mol. Ther. *18*, 1357–1364.
27. Aarsland, D., Rongve, A., Nore, S.P., Skogseth, R., Skulstad, S., Ehrt, U., Hoprekstad, D., and Ballard, C. (2008). Frequency and case identification of dementia with Lewy bodies using the revised consensus criteria. Dement. Geriatr. Cogn. Disord *26*, 445–452.
28. Jayaraman, M., Ansell, S.M., Mui, B.L., Tam, Y.K., Chen, J., Du, X., Butler, D., Eltepu, L., Matsuda, S., Narayanannair, J.K., et al. (2012). Maximizing the potency of siRNA lipid nanoparticles for hepatic gene silencing in vivo. Angew. Chem. Int. Ed. Engl. *51*, 8529–8533.
29. Kaji, S., Maki, T., Kinoshita, H., Uemura, N., Ayaki, T., Kawamoto, Y., Furuta, T., Urushitani, M., Hasegawa, M., Kinoshita, Y., et al. (2018). Pathological Endogenous α-Synuclein Accumulation in Oligodendrocyte Precursor Cells Potentially Induces Inclusions in Multiple System Atrophy. Stem Cell Report. *10*, 356–365.
30. Kaji, S., Maki, T., Ueda, J., Ishimoto, T., Inoue, Y., Yasuda, K., Sawamura, M., Hikawa, R., Ayaki, T., Yamakado, H., and Takahashi, R. (2020). BCAS1-positive immature oligodendrocytes are affected by the α-synuclein-induced pathology of multiple system atrophy. Acta Neuropathol. Commun. *8*, 120.
31. Maki, T., Maeda, M., Uemura, M., Lo, E.K., Terasaki, Y., Liang, A.C., Shindo, A., Choi, Y.K., Taguchi, A., Matsuyama, T., et al. (2015). Potential interactions between

pericytes and oligodendrocyte precursor cells in perivascular regions of cerebral white matter. Neurosci. Lett. *597*, 164–169.

32. Hoshino, T., Yamakado, H., Takahashi, R., and Matsuzawa, S.I. (2020). Susceptibility to erastin-induced ferroptosis decreases during maturation in a human oligodendrocyte cell line. FEBS Open Bio. *10*, 1758–1764.
33. Shen, J.S., Watabe, K., Ohashi, T., and Eto, Y. (2001). Intraventricular administration of recombinant adenovirus to neonatal twitcher mouse leads to clinicopathological improvements. Gene Ther. *8*, 1081–1087.
34. von Gerichten, J., Schlosser, K., Lamprecht, D., Morace, I., Eckhardt, M., Wachten, D., Jennemann, R., Gröne, H.J., Mack, M., and Sandhoff, R. (2017). Diastereomer-specific quantification of bioactive hexosylceramides from bacteria and mammals. J. Lipid Res. *58*, 1247–1258.
35. Akiyama, H., Ide, M., Nagatsuka, Y., Sayano, T., Nakanishi, E., Uemura, N., Yuyama, K., Yamaguchi, Y., Kamiguchi, H., Takahashi, R., et al. (2020). Glucocerebrosidases catalyze a transgalactosylation reaction that yields a newly-identified brain sterol metabolite, galactosylated cholesterol. J. Biol. Chem. *295*, 5257–5277.
36. Ikuno, M., Yamakado, H., Akiyama, H., Parajuli, L.K., Taguchi, K., Hara, J., Uemura, N., Hatanaka, Y., Higaki, K., Ohno, K., et al. (2019). GBA haploinsufficiency accelerates alpha-synuclein pathology with altered lipid metabolism in a prodromal model of Parkinson's disease. Hum. Mol. Genet. *28*, 1894–1904.
37. Nakanishi, E., Uemura, N., Akiyama, H., Kinoshita, M., Masanori, S., Taruno, Y., Yamakado, H., Matsuzawa, S.I., Takeda, S., Hirabayashi, Y., and Takahashi, R. (2021). Impact of Gba2 on neuronopathic Gaucher's disease and α-synuclein accumulation in medaka (Oryzias latipes). Mol. Brain *14*, 80.

**OMTN, Volume *35***

## Supplemental information

# A lipid nanoparticle-based oligodendrocyte-specific mRNA therapy

**Masanori Sawamura, Kiyoshi Tachikawa, Rie Hikawa, Hisako Akiyama, Seiji Kaji, Ken Yasuda, Angel I. Leu, Hyojung Hong, Rajesh Mukthavaram, Pad Chivukula, Hodaka Yamakado, Yoshio Hirabayashi, Ryosuke Takahashi, and Shu-ichi Matsuzawa**

**Table S1. Primer sequences used for qPCR**

| Gene | Forward primer | Reverse primer |
|---|---|---|
| *GAPDH* | GGT CGG AGT CAA CGG ATT TG | TCA GCC TTG ACG GTG CCA TG |
| *LDLR* | GAG AGC TTG TGC CGA GAT GTG | CCG CAG TTG TTA GTG CCA TCA |
| *VLDLR* | AGA AAA GCC AAA TGT GAA CCC T | CAC TGC CGT CAA CAC AGT CT |
| *ApoER2* | ATG AGT GAC GTG AAT CCA CCC | GTC CAG GGC GGA ATA TGA GAA |

**Movies S1. LUNAR® transfers EGFP mRNA in oligodendrocyte.**

After treatment with LUNAR®-*EGFP* mRNA, EGFP is gradually expressed in oligodendrocytes.

**Movies S2. LUNAR® does not transfers EGFP mRNA in neurons.**

After treatment with LUNAR®-*EGFP* mRNA, EGFP is not expressed in neurons.

**Movies S3. The motor phenotype of non-injected twitcher mice.**

The non-injected twitcher mice present motor dysfunction and tremorous movements.

**Movies S4. The motor phenotype of twitcher mice treated with LUNAR®-*GalC* mRNA.**

LUNAR®-*GalC* mRNA ameliorates the motor abnormalities in twitcher mice.
